# Inguinal Aneurism Cured by Digital Compression

**Published:** 1860-10

**Authors:** W. C. Nichols


*Inguinal Aneurism Cured by Digital Compression:* Reported by W. C. Nichols, M. D.

Having a wish, during the past winter, to observe the treatment adopted by Dr. Warren Stone in various surgical affections, I frequently visited the wards in his charge at the Charity Hospital. Entering one of these on the 15th of February, ultimo, my attention was directed to a nurse who was attempting to adjust a compress to the groin of a patient in bed.

This patient informed me that his leg grew painful, and began to swell in January of the present year, and that a diversity of applications had been made to the tumid member by persons ignorant of his condition.

On the 2d of February, 1860, he consulted Dr. Andrew Smyth, at that time Assistant Surgeon of the Charity Hospital, who readily appreciating the cause of his malady, referred him to Dr. Stone for treatment.

I found, on examination, a pulsating tumor, about the size of a goose's egg, situated in the right groin above Poupart's ligament. The hand applied over the course of the external iliac artery received an impulse and a thrill characteristic of arterial tumors, whilst auscultation recognized a distinct aneurismal murmur. In point of fact, as each contraction of the heart sent a wave of blood along the artery, the heaving pulsation of the tumor was evident to inspection. The patient complained of great pain in the tumor when the blood rushed freely into it, and apprehensive of torture, he was eager to break the force of the current by digital or instrumental compression. By thrusting a thumb or finger deeply into the iliac region along the course of the artery, the painful mass could be circumscribed, and the flow of blood into the sac could be easily arrested. The superficial vessels of the corresponding leg and thigh, owing to the pressure of the aneurism, were tortuous, whilst the member was swollen and of a livid color. Satisfied that all pulsation in the tumor could be checked by pressing the thumb on the course of the artery, I felt assured that by securing competent assistants, the flow of blood into the aneurism could be moderated a sufficient length of time to promote the fibrination of its contents and effect a cure.

With the permission of Dr. Stone, I undertook the treatment of this case by digital compression. The rapidity of this cure must be attributed, in great part, to the intelligence of the gentlemen, both physicians and students, who kindly afforded assistance; for imbued with enthusiasm in their profession, they were ever vigilant in adopting such measures as would facilitate recovery and conduce to the comfort of the patient. I subjoin their names: Drs. Roberts and Fournier; Mess. Murphy, Tate, Turner, Shields, Robertson, Ford, Richardson, Parker, Jackson, McGregor, Lewis, Jones, Campbell, Denman, Collins, Moody, Profilet, Stickney, Sawyer, Haynes, Duffel, and Burke.

This patient, named Patrick Gilday, a native of Ireland, aged 28, was admitted to the Charity Hospital on the second of February, 1860.

Digital compression was begun in this case at five minutes after five o'clock, P, M., on Friday, February 17th, 1860.—The treatment was begun by thrusting the thumb directly against the neck of the sac, so that all pulsation was speedily arrested. We at first found much difficulty in accomplishing our purpose; for the tenderness of the parts produced spasm and rigidity of the abdominal muscles, and this condition, conjoined with the alternate elevation and depression of the abdominal walls, rendered doubtful our ultimate success. The indication was to benumb the sensibility of the patient, and a draught containing half a grain of morphia was administered. The benign influence of ihis potion rendered compression tolerable, and we entered upon our night watch with hopes of success.

On Saturday moruing, February 18th. the tumor had diminished in size, and the pulsations were less forcible. The most flattering indications of recovery were presented during the day; for the patient, buoyed with hopes of relief, and calmed by the opiates, submitted without a murmur to the treatment. During the afternoon, when each assistant gave place to his successor, we discovered that pulsation would be arrested for several seconds; and at eleven, P. M., pulsation entirely disappeared. We continoed pressure during the remainder of Saturday night, and on Sunday morning, February 19th, at nine o'clock, A. M., during Dr. Stone's customary visit to the ward, he pronounced the cure effectual, and advised the withdrawal of pressure. Surprised at the rapidity with which this sac was obliterated, and fearful of untoward accidents, I requested the students to remain with the patient during the day. They continued their watch till eleven, P. M., only giving occasional pressure, and then retired, confident of a triumph in conservative surgery.

Thus, after making presure for *thirty hours*, all pulsation ceased; and within *forty hours* the cure was announced as successful, and within *fifty-four hours* our watch over the patient was withheld.

The patient was not permitted to leave his bed for several days; yet, excepting a bandage applied to his leg to counteract œdema, no futher treatment was directed. This patient left the Hospital on the 29th of February, 1860. He has returned on two occasions, submitting himself to inspection; and on May 3d, he came, by request, to the Hospital, exhibiting a leg free from enlargement, yet presenting about the ankle a more livid hue than the opposite member. The aneurismal tumor had contracted to a firm nucleus about the size of a walnut. The patient informed me that since his departure from the Hospital, he has continued to work at a cotton press, around which every laborer is required to undergo great muscular exertion. It is a matter of surprise that no ill consequences have ensued.

In glancing over the records of surgery, I am unable to find a parallel instance of success in treating inguinal aneurism by digital compression. The cases reported by Broca, Michoux, Vanzetti, Gross, and others, were confined to the extremities; and I am not aware that any surgeon has hitherto succeeded in controlling an aneurism by digital compression applied so near the great centre of the circulation. Dr. Gross, in his treatise on Surgery (1859), estimating the value of digital compression as compared with other modes of treatment, remarks: "It is to aneurisms of the extremities that this procedure is mainly, if not exclusively applicable, as the compresion must be made to bear upon some point of the principal artery of the limb." And he adds: "The external iliac has been subjected to the same procedure in two cases of inguinal aneurism; in one, the pressure was unbearable, and in the other, the assistants became so fatigued that it was discontinued. Moreover, it is very difficult in this situation to keep up the pressure, and such cases should, therefore, be excluded."

Satisfied, from the knowledge acquired in the treatment of this aneurism, that digital compression should be more frequently resorted to by surgeons, and hoping to invite attention to this mode of managing such maladies, I willingly submit this report for publication. Should failure ensue with this procedure in some cases of aneurism, its use prior to more serious operations, must necessarily protect the patient against many untoward accidents consequent on the sudden arrest of the nutritive current to an important organ.—*N. O. Med. & Surg. Journal.*

